# The Ratios of CD8^+^ T Cells to CD4^+^CD25^+^ FOXP3^+^ and FOXP3^-^ T Cells Correlate with Poor Clinical Outcome in Human Serous Ovarian Cancer

**DOI:** 10.1371/journal.pone.0080063

**Published:** 2013-11-14

**Authors:** Claudia C. Preston, Matthew J. Maurer, Ann L. Oberg, Daniel W. Visscher, Kimberly R. Kalli, Lynn C. Hartmann, Ellen L. Goode, Keith L. Knutson

**Affiliations:** 1 Department of Immunology, Mayo Clinic, Rochester, Minnesota, United States of America; 2 Department of Health Science Research, Mayo Clinic, Rochester, Minnesota, United States of America; 3 Division of Anatomic Pathology, Mayo Clinic, Rochester, Minnesota, United States of America; 4 Division of Medical Oncology, Mayo Clinic, Rochester, Minnesota, United States of America; 5 Vaccine and Gene Therapy Institute, Port St. Lucie, Florida, United States of America; Université Libre de Bruxelles, Belgium

## Abstract

Ovarian cancer is an immune reactive malignancy with a complex immune suppressive network that blunts successful immune eradication. This suppressive microenvironment may be mediated by recruitment or induction of CD4^+^ regulatory T cells (Tregs). Our study sought to investigate the association of tumor-infiltrating CD4^+^CD25^+^FOXP3^+^ Tregs, and other immune factors, with clinical outcome in serous ovarian cancer patients. We performed immunofluorescence and quantification of intraepithelial tumor-infiltrating triple positive Tregs (CD4^+^CD25^+^FOXP3^+^), as well as CD4^+^CD25^+^FOXP3^-^, CD3^+^ and CD8^+^ T cells in tumor specimens from 52 patients with high stage serous ovarian carcinoma. Thirty-one of the patients had good survival (i.e. > 60 months) and 21 had poor survival of < 18 months. Total cell counts as well as cell ratios were compared among these two outcome groups. The total numbers of CD4^+^CD25^+^FOXP3^+^ Tregs, CD4^+^CD25^+^FOXP3^-^, CD3^+^ and CD8^+^ cells were not significantly different between the groups. However, higher ratios of CD8^+^/CD4^+^CD25^+^FOXP3^+^ Treg, CD8^+^/CD4^+^ and CD8/CD4^+^CD25^+^FOXP3^-^ cells were seen in the good outcome group when compared to the patients with poor outcome. These data show for the first time that the ratios of CD8^+^ to both CD4^+^CD25^+^FOXP3^+^ Tregs and CD4^+^CD25^+^FOXP3^-^ T cells are associated with disease outcome in ovarian cancer. The association being apparent in ratios rather than absolute count of T cells suggests that the effector/suppressor ratio may be a more important indicator of outcome than individual cell count. Thus, immunotherapy strategies that modify the ratio of CD4^+^CD25^+^FOXP3^+^ Tregs or CD4^+^CD25^+^FOXP3^-^ T cells to CD8^+^ effector cells may be useful in improving outcomes in ovarian cancer.

## Introduction

Ovarian cancer has the highest mortality rate of cancers exclusive to women. Despite many therapeutic efforts utilizing new chemotherapies, the cure rate has not improved substantially in decades [1–3]. It is well known that clinical outcomes in ovarian cancer are quite heterogeneous and not easily predicted by standard clinical and pathologic characteristics (e.g., grade, tumor histology) [4–6]. This suggests that there may be other tumor microenvironment or host characteristics with a dominant role in survival. In recent years, there has been interest in understanding the role of the patient’s immune response to her ovarian cancer, as the disease is thought to be a naturally immune reactive malignancy with a complex suppressive network that effectively blunts successful immune eradication. Over the past decade, studies have demonstrated the importance of the immune system in affecting patient outcome. Notably, Zhang and colleagues published a study that showed that the majority of ovarian cancer patients had tumor-infiltrating CD3^+^ T cells and that infiltration was positively associated with survival [7]. The presence of T cells was particularly beneficial for those individuals who demonstrated a complete clinical response to surgery and chemotherapy in which the five-year survival was 74% compared to 12% for those without T cells. Subsequent studies have refined our understanding of intra-tumoral T cells, such as the work by Sato et al., that showed patients who had high levels of infiltrating cytotoxic T lymphocytes (CTL) had a median survival of 55 months versus those with few or no CTL who had a survival of 26 months [8]. A unique feature of the conventional therapeutic strategies for ovarian cancer is that T cell function is rapidly recovered following conventional chemotherapy [9]. The antigens to which the patients are naturally responding are now being systematically studied [10,11]. Collectively, these findings show that anti-tumor immunity is elicited against ovarian cancers and impacts the clinical course of the disease. However, it is now apparent that the anti-tumor immunity is counterbalanced by an immune suppressive microenvironment [7,12–15]. 

One of the cellular factors that can mediate this immune suppression in the tumor microenvironment is the regulatory T cell (Treg) population. Research on Tregs has advanced rapidly, especially in context of cancer. Tregs are a heterogeneous CD4^+^ T cell subpopulation whose primary function is immune regulation by blocking the function of activated T cells. CD4^+^ Tregs can be divided into two main subsets: naturally occurring Tregs with a CD4^+^CD25^+^FOXP3^+^ phenotype and induced Tregs with a variable CD25 expression [12]. Previous studies have shown that forkhead box P3 (FOXP3) is a central transcription factor that regulates the development and function of CD4^+^ Tregs [16,17]. Moreover, studies have also demonstrated that expression of CD25, a component of the high-affinity IL-2 receptor, is essential for Treg survival, which is highly dependent on IL-2 [18,19]. In the past decade there have been several studies linking Tregs with survival in many types of cancer. In ovarian cancer, Curiel and colleagues initially showed a strong association of CD4^+^CD25^+^ T cells with poor survival [14]. Of note, the specific role of triple stained CD4^+^CD25^+^FOXP3^+^ T cells was not assessed. A few years later, Sato and colleagues failed to see any direct association of CD25^+^FOXP3^+^ T cells in ovarian cancer, but did show that low total CD8^+^ T cell counts and the CD8^+^/CD25^+^FOXP3^+^ T cell ratio were associated with poorer survival, again in a population with varying histologies [8]. In yet a more recent study, Milne and colleagues showed, in high grade serous ovarian cancer, that the count of intraepithelial cells singly stained for FOXP3^+^ was associated with greatly improved survival [20]. However, their study did not specifically define the cell type expressing FOXP3. Given that other cells in addition to T cells express FOXP3 (e.g., tumor cells and B cells), the significance of that work, while interesting, remains unclear [21,22]. Furthermore, since both CD8^+^ and CD4^+^, regulatory and *in vitro* activated T cells are known to express FOXP3, the specific identity of the cell type involved in modifying survival is unknown. However, more recently, Kryczek and colleagues showed that in primary tumors or autoimmune lesions, FOXP3 and CD25 is a highly specific and reliable marker set for primary human CD4^+^ Tregs [23]. 

The main focus of our study was to examine the association of tumor infiltrating CD4^+^CD25^+^FOXP3^+^ Tregs and other T cells with clinical outcome in epithelial ovarian cancer patients, since this approach of triple staining has not been done in ovarian tumors to the best of our knowledge. Specifically, we studied a unique cohort, limited to patients with advanced stage serous ovarian cancer (the most common and most lethal histology), comprised of two subgroups of patients with widely divergent clinical outcomes, one with very poor survival (< 18 months) and the other one of patients with much better survival (> 60 months).

## Materials and Methods

### Patients and clinical characteristics

The study was approved by the Mayo Clinic Institutional Review Board and all patients gave written informed consent for research use of specimens. Power calculations were done via simulation to estimate the number of samples needed to detect a meaningful survival difference in patients with ovarian cancer. Fifty-two patients, chosen from disparate outcome groups with poor outcome (< 18 month survival) and good outcome (> 60 month survival), provided 82.5% power at an alpha of 0.05 to detect an overall survival hazard ratio of 1.65 between dichotomous CD4^+^CD25^+^FOXP3^+^ Treg groupings across all ovarian cancer patients. This translates to at least 80% power to detect an effect size (change in number or ratio of CD4^+^CD25^+^FOXP3^+^ Tregs) of 0.8 when evaluating Tregs between the two disparate outcome groups with poor outcome (*n*=21) and good outcome (*n*=31) with a Kruskall Wallace test, as performed in this study. All samples were selected from patients with optimally debulked, high stage, serous ovarian, fallopian tube or primary peritoneal carcinoma.

### Staining and immunofluorescence analysis of tissue specimens

All frozen specimen blocks, previously embedded in Tissue-Tek OCT (Sakura Finetek, Torrance, CA), were cryosectioned (5µm), fixed in acetone for 10 minutes, air dried for 1 hour and store at -80°C until use. Before staining, all slides were blocked for 10 minutes at room temperature (25°C) with serum-free protein block (Dako, Carpinteria, CA) and then washed with phosphate buffered saline (PBS). Staining was performed by incubation of the slides at room temperature (25°C) in moist dark chambers for 2 hours with primary antibodies diluted in antibody diluent reagent (Dako, Carpinteria, CA). Rabbit polyclonal anti-human FOXP3 (Abcam, Cambridge, MA) was used at a dilution 1:75, mouse monoclonal anti-human CD4 (Abcam, Cambridge, MA) at 1:100, rat monoclonal anti-human CD25 (Abd Serotec, Raleigh, NC) at 1:100, mouse monoclonal anti-human CD8 (BD Pharmingen, San Diego, CA) at 1:100, and mouse monoclonal anti-human CD3 (BD Pharmingen, San Diego, CA) at 1:50. Following staining with primary antibodies, slides were then washed for 15 minutes with PBS and incubated for 1 hour in a moist dark chamber with secondary antibodies (Alexa fluor 405, 488 and 594 conjugated, Invitrogen, Life Technologies, Grand Island, NY) at a dilution of 1:500. Antibodies for FOXP3, CD4 and CD25 were used for triple staining of Tregs while antibodies for CD3 and CD8 were used as single stains. Singly stained cells were also counterstained with 4’,6-diamidino-2-phenylindole (DAPI, 100 ng/ml) for 30 minutes. Human tonsillar tissue was used as a positive control and negative controls were done by omitting the primary antibody and using isotype controls for each antibody. 

### Confocal microscopy and cell quantification

The stained slides were evaluated in an LSM 510 confocal laser scanning microscope (Carl Zeiss MicroImaging, Inc., Oberkochen, Germany). Intraepithelial stained cells were assessed on high-powered magnification scanned fields of random epithelial sections avoiding stromal areas. The scanned fields were spaced to avoid overlap and bleaching and to cover the entire tumor section. Twenty random fields (300 µm^2^) were digitally photographed for each patient sample. Quantification of positive cells was performed manually by observers blinded to all clinical information. A subset of randomly selected samples were reviewed by a secondary observer. Total counts of cells from each field were recorded in either the triple stained (CD4, CD25 and FOXP3) or single stained (CD8 or CD3) samples. 

When tumor samples were examined grossly under confocal microscopy, tumor-infiltrating lymphocytes were observed in both the stroma and the epithelium. Thus, careful attention was paid to establishing the counting fields exclusively within epithelial areas. The following cells were quantified in the triple stained samples and used in the analysis: CD4^+^CD25^+^FOXP3^+^, CD4^+^CD25^+^FOXP3^-^, CD4^+^CD25^-^FOXP3^-^, CD4^+^CD25^-^FOXP3^+^, CD4^-^CD25^+^FOXP3^+^, CD8^+^DAPI^+^ and CD3^+^DAPI^+^. Intraepithelial tumor infiltrating Tregs were defined as CD4^+^CD25^+^FOXP3^+^ cells, with CD4 (red signal) and CD25 (green signal) stains detected in the cytosolic and membrane areas giving an orange and/or yellow pattern and FOXP3 (blue signal) detected as an intra-nuclear and typically dotted stain pattern. The primary assessment of CD4^+^CD25^+^FOXP3^+^ Treg quantification was the total Treg count across all 20 pictures from a tumor sample. This was done in contrast to previous studies which generally quantified Tregs using user-identified focal areas of infiltration [8,14]. We tested the validity of these prior approaches by also using secondary measurements for each tumor, namely the highest single image count from a tumor sample (MAX1) and the sum of the three highest counts from a tumor sample (MAX3). 

### Tumor-infiltrating lymphocyte isolation

In further investigation, tumor-infiltrating lymphocytes (TILs) were harvested from six ovarian tumors and isolated by discontinuous Ficoll gradient as previously described [24]. Briefly, the lymphocytes were separated from the tumor cells by centrifugation of the cell suspension on a two layer Ficoll gradient, 100% layer on the bottom and 75% layer on top. The isolated TILs were then stained for flow cytometric analysis as described below.

### Flow cytometry

Cell surface and intracellular marker staining was performed in isolated TILs (1 x 10^6^) as described previously [25]. Two sets of experiments were performed; in the first set un-stimulated cells were stained with the following conjugated antibodies: CD3-PE-Cy7, CD4-Pacific Blue, CD8-Alexa Fluor 700, CD1c-APC, BDCA2-FITC and CD68-PerCP-Cy5.5.  In the second, we set stained un-stimulated and stimulated cells for CD4-Pacific blue, CD25-PE, FOXP3-Alexa Fluor 647 and TGF-β-PE-Cy7.  Prior to staining TILs were stimulated by incubation with 5μg/ml concanavalin-A (Con-A, Sigma-Aldrich, St Louis, MO) and 0.7μl/ml BD GolgiStop™ (BD Bioscience, San Jose, CA) for 8 hours, then washed, counted and resuspended in FACS buffer (PBS with 1mM/L EDTA and 3% bovine serum albumin).  FOXP3 and cytokine staining was carried out according to the manufacturer’s intracellular staining protocol (eBioscience, San Diego, CA).  Appropriate isotype antibodies were used as controls.  All antibodies were purchased from BD Bioscience (San Jose, CA) except for the following antibodies: TGF-β-PE-Cy7 and CD3-PE-Cy7 were from eBioscience (San Diego, CA), BDCA2-FITC from Miltenyi Biotec (Auburn, CA) and CD68-PerCP-Cy5.5 was acquired from BioLegend (San Diego, CA).  Samples were run on a FACS Scan II and analyzed using FlowJo 10.0.5.

### Data analysis

The distributions of CD4^+^CD25^+^FOXP3^+^ Treg counts between patients with good and poor outcome were examined graphically and compared using Wilcoxon rank-sum tests (Mann–Whitney *U* test). Ratios were calculated as the number of CD8^+^ cells divided by the number of CD4^+^CD25^+^FOXP3^+^ Tregs for a given patient. Cell counts are summarized as median and interquartile range (IQR). For pairwise comparisons of flow cytometry data, a paired Student’s T test was used. The statistical significance was set at a p-value of ≤ 0.05.

## Results

### Clinical patient characteristics

Fifty two tumor samples meeting study criteria were selected from the tissue bank. Patient characteristics are summarized in Table 1. The median age of patients in the study was 65 years (range, 37-86 years). All patients were diagnosed with advanced stage (71% stage III, 29% stage IV) disease, were optimally debulked, and had tumors with serous morphology. Median survival in the poor outcome group was 12.4 months (range 3.0-16.7) and median survival in the good outcome group has not yet been reached with 73.8 months median follow-up (range 60.1-116.8). 

**Table 1:** Patient Characteristics.

	Good Outcome	Poor Outcome	
	(*n*=31)	(*n*=21)	P-value
**Age at Diagnosis**			
Median	62.0	69.0	0.0059
Range	(37.0-80.0)	(50.0-86.0)	
**Stage**			
III	23 (74.2%)	14 (66.7%)	0.56
IV	8 (25.8%)	7 (33.3%)	
**Grade**			
Low	3 (9.7%)	0 (0.0%)	0.20
High	28 (90.3%)	21 (100%)	
**Vital Status**			
Alive	24 (77.4%)	0 (0.0%)	N.A.
Dead	7 (22.6%)	21 (100.0%)	
**First Line Chemotherapy**			
Platinum	2 (6.5%)	1 (4.8%)	0.20
Platinum and Taxane	27 (87.1%)	15 (71.4%)	
Other	2 (6.5%)	5 (23.8%)	

Good outcome > 60 month survival, poor outcome < 18 month survival; all cases were serous cancers and optimally cytoreduced.

### Enumeration of intraepithelial tumor-infiltrating T cells

**Figure 1:**
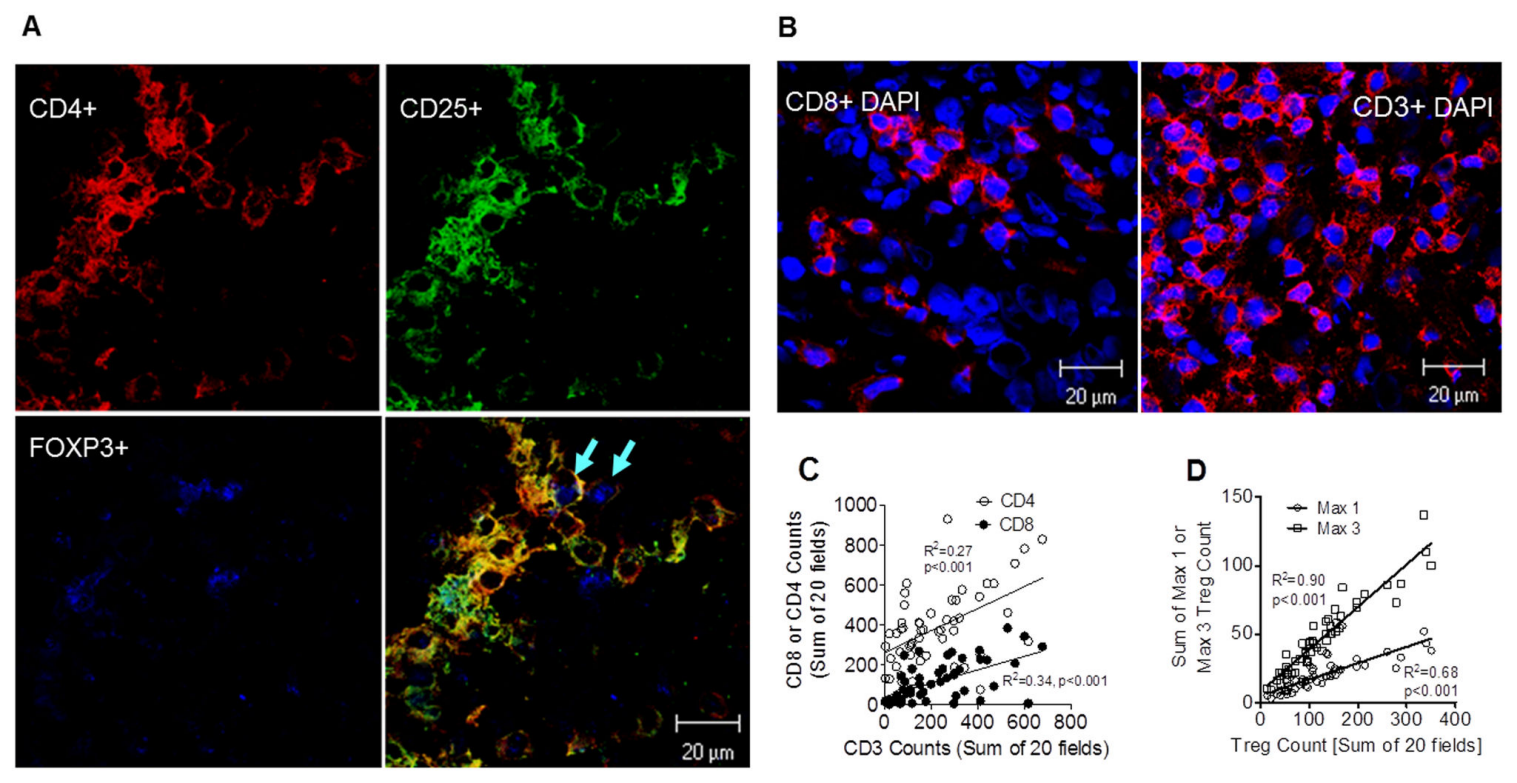
Triple-positive Tregs infiltrate into the tumor microenvironment of ovarian cancer. (**A**) Photo of CD4^+^CD25^+^FOXP3^+^ Tregs in ovarian tumor samples. Top left panel shows CD4 expression (red signal), top right panel shows CD25 expression (green signal), bottom left panel shows FOXP3 expression (blue signal) and bottom right panel shows the combined signals with arrows pointing to triple stained CD4^+^CD25^+^FOXP3^+^ Tregs. In this picture Tregs are seen in close contact to CD4^+^CD25^+^FOXP3^-^ cells. (**B**) Photo of intraepithelial infiltrating CD8^+^ cytotoxic T cells (left panel) and CD3^+^ lymphocytes (right panel) in ovarian cancer. This representative scan shows CD8 and CD3 expression (red signal = CD8 or CD3, blue signal = DAPI) in ovarian tumor mass. (**C**) Correlation plot comparing CD3 counts to either CD8 (filled symbols) and CD4 counts (open symbols). The symbols represent the sum of the 20 fields counted for a unique patient. All patients are represented. The inset lines are the product of linear regression analysis. (**D**) Shows the overall mean (± SEM, *n*=52) CD3^+^, CD4^+^, and CD8^+^ T cell counts (sum of 20 fields) for all patients. (**E**) Correlation plot comparing the total Treg (i.e. CD4^+^CD25^+^Foxp3^+^) counts with the sum of the maximum 3 (MAX3) fields or the maximum field count (MAX1). The symbols represent the sum of the 20 fields counted for a unique patient. All patients are represented. The inset lines are least squares regression lines.

Intraepithelial infiltrating cells (CD4^+^CD25^+^FOXP3^+^ Tregs, CD4^+^, CD8^+^, CD3^+^, CD4^+^CD25^+^FOXP3^-^ and CD4^+^CD25^-^FOXP3^+^) were quantified and analyzed in all fields. Representative scanned fields of tumor infiltrating putative CD4^+^CD25^+^FOXP3^+^ Tregs as well as CD4^+^CD25^+^FOXP3^-^ are shown in Figure 1A. Figure 1B shows representative CD8^+^ and CD3^+^ T cell infiltration. Analysis revealed a modest but statistically significant linear correlation between counts of CD3^+^ T cells and the CD4^+^ or CD8^+^ T cells (Figure 1C). 

Given that prior studies examined and enumerated non-random T cell enriched fields, we examined the validity of this approach by assessing the correlation between the total CD4^+^CD25^+^FOXP3^+^ Treg count across all 20 fields and the MAX1 and MAX3 counts. As shown in Figure 1D, there were very strong statistically significant correlations, suggesting that prior approaches to enumerating infiltrating T cells are largely valid.

### T cell counts did not directly correlate with clinical outcome in human serous ovarian cancer

Initial analyses focused on correlation of cell counts with outcome groups. In contrast to prior work, intraepithelial CD3^+^ T cells were not observed at significantly different levels in patients with good outcome (median 154 cells (sum of 20 fields), IQR 89-297) compared with patients with poor outcome (median 179, IQR 73-333, Table 2). Similarly, the quantities of infiltrating CD4^+^ (370, IQR 249-461 in the good group vs. 377, IQR 264-524 in the poor group) and CD8^+^ T cells (115, IQR 53-180 in the good group vs. 48, IQR 17-180 in the poor group) were not significantly different between the good and poor outcome groups. 

**Table 2:** Comparisons of T cell levels or ratios.

	Good Outcome (n=31)		Poor Outcome (n=21)		
	Median	IQR	Median	IQR	P-value
**Cell Counts^1^**					
CD3^+^	154	89-297	179	73-333	0.881
CD4^+^	370	249-461	377	264-524	0.514
CD8^+^	115	53-180	48	17-180	0.271
CD4^+^CD25^+^FOXP3^+^	101	55-161	109	92-146	0.444
CD4^+^CD25^+^FOXP3^-^	148	90-187	147	105-231	0.251
**Ratios^2^**					
CD8^+^/CD3^+^	0.58	0.34-0.91	0.43	0.20-0.70	0.198
CD4^+^/CD3^+^	2.06	1.33-3.43	1.73	1.32-3.67	0.985
CD8^+^/CD4^+^	**0.27**	**0.20-0.44**	**0.13**	**0.07-0.37**	**0.050**
CD3^+^/ CD4^+^CD25^+^FOXP3^+^	1.72	0.95-2.68	1.38	0.79-2.49	0.695
CD3^+^/ CD4^+^CD25^+^FOXP3^-^	1.31	0.71-2.06	1.24	0.72-1.64	0.490
CD8^+^/ CD4^+^CD25^+^FOXP3^+^	**0.98**	**0.49-1.79**	**0.32**	**0.25-1.02**	**0.027**
CD8^+^/ CD4^+^CD25^+^FOXP3^-^	**0.65**	**0.43-1.19**	**0.46**	**0.13-0.77**	**0.048**
CD4^+^/ CD4^+^CD25^+^FOXP3^+^	2.91	2.59-4.31	3.04	2.39-3.46	0.332
CD4^+^/ CD4^+^CD25^+^FOXP3^-^	2.50	1.94-3.19	2.16	1.80-2.64	0.351

^1^Sum of cell counts of all 20 fields, ^2^Ratio of the sum of all 20 fields; IQR, interquartile range; statistically significant changes are shown in bold.

Using the CD25 and FOXP3 markers, CD4^+^ T cells could be divided into four groups. The two major groups were CD4^+^CD25^+^FOXP3^+^ Tregs (~33.2% among all patients) and CD4^+^CD25^+^FOXP3^-^ T cells (~43.1%). The two other minor groups were the CD4^+^CD25^-^FOXP3^+^ T cells (~14.4%) and CD4^+^CD25^-^FOXP3^-^ T cells (~9.3%). We saw no difference in the levels of CD4^+^CD25^+^FOXP3^-^ T cells between patient outcome groups. The median levels were 148 cells (IQR 90-187) in the good outcome group and 147 (IQR 105-231) in the poor outcome group (Table 2). Similarly, the median counts of CD4^+^CD25^+^FOXP3^+^ Tregs among patients with good outcome were not different compared to the patients with poor outcome. All tumor samples showed CD4^+^CD25^+^FOXP3^+^ Treg infiltration with a combined range of 14-350 cells. The percentage of CD4^+^CD25^+^FOXP3^+^ Tregs among the total intraepithelial CD4^+^ infiltrating cells was 31 ± 10% in the good outcome group compared to 34 ± 11% in the poor outcome group of patients (p=0.319). 

**Figure 2:**
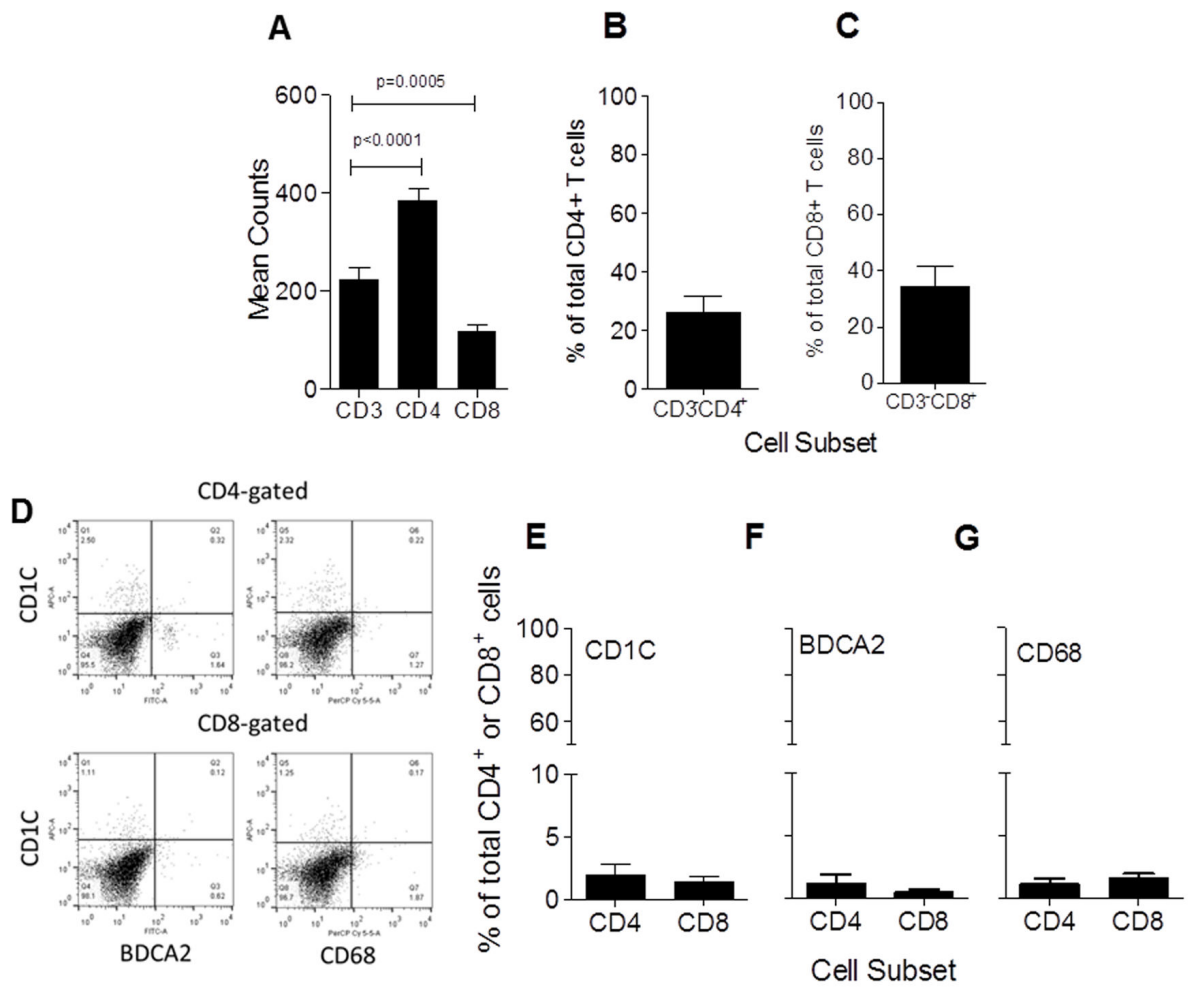
CD4^+^ or CD8^+^ myeloid cells contribute are a minor fraction of the total CD4^+^ or CD8^+^ cells, respectively. (**A**-**B**) Bar graphs showing the mean (± SEM, *n*=3) levels of CD3-CD4^+^ or CD8^+^, respectively as a percentage of the total CD4^+^ or CD8^+^ T cells, respectively. (**C**) shown are 4 representative dual color dot plots staining either CD4- or CD8-gated cells. The antibody specificities are noted with the axis. (**D**) Shown are the mean (± SEM, *n*=3) levels of CD11C^+^, BDCA2^+^ and CD68^+^ cells as a total CD4^+^ or CD8^+^ cells (X-axis).

One notable observation from these comparisons is that the CD4^+^ T cell counts were higher than the CD3^+^ counts when equivalence was expected. (Figures 2A). One potential reason for the lack of correlation could be that CD4 and CD8 antibodies are staining a mixture of both lymphoid and myeloid cells, since it has been previously reported that both markers can be expressed on various leukocyte subsets including lymphoid and myeloid. Alternatively, CD3 could be down-regulated in T cells due to chronic stimulation in the tumor microenvironment [26,27]. Considering these issues, we purified tumor infiltrating mononuclear cells from three patients with ovarian cancer, stained them with various lymphoid and myeloid markers and performed flow cytometric analysis. Staining revealed that 26 ± 3% (n=3, SEM) and 34 ± 4% of CD4^+^ and CD8^+^ cells, respectively are CD3 negative (Figures 2B-C). The analysis of CD11c^+^, BDCA2^+^, and CD68^+^ cells suggests that myeloid DC, plasmacytoid DC and macrophages together constitute less than a total of 5% of each of the CD4^+^ and CD8^+^ cells in ovarian tumors (Figures 2D-G).

### T cell ratios correlate with clinical outcome in human serous ovarian cancer

**Figure 3:**
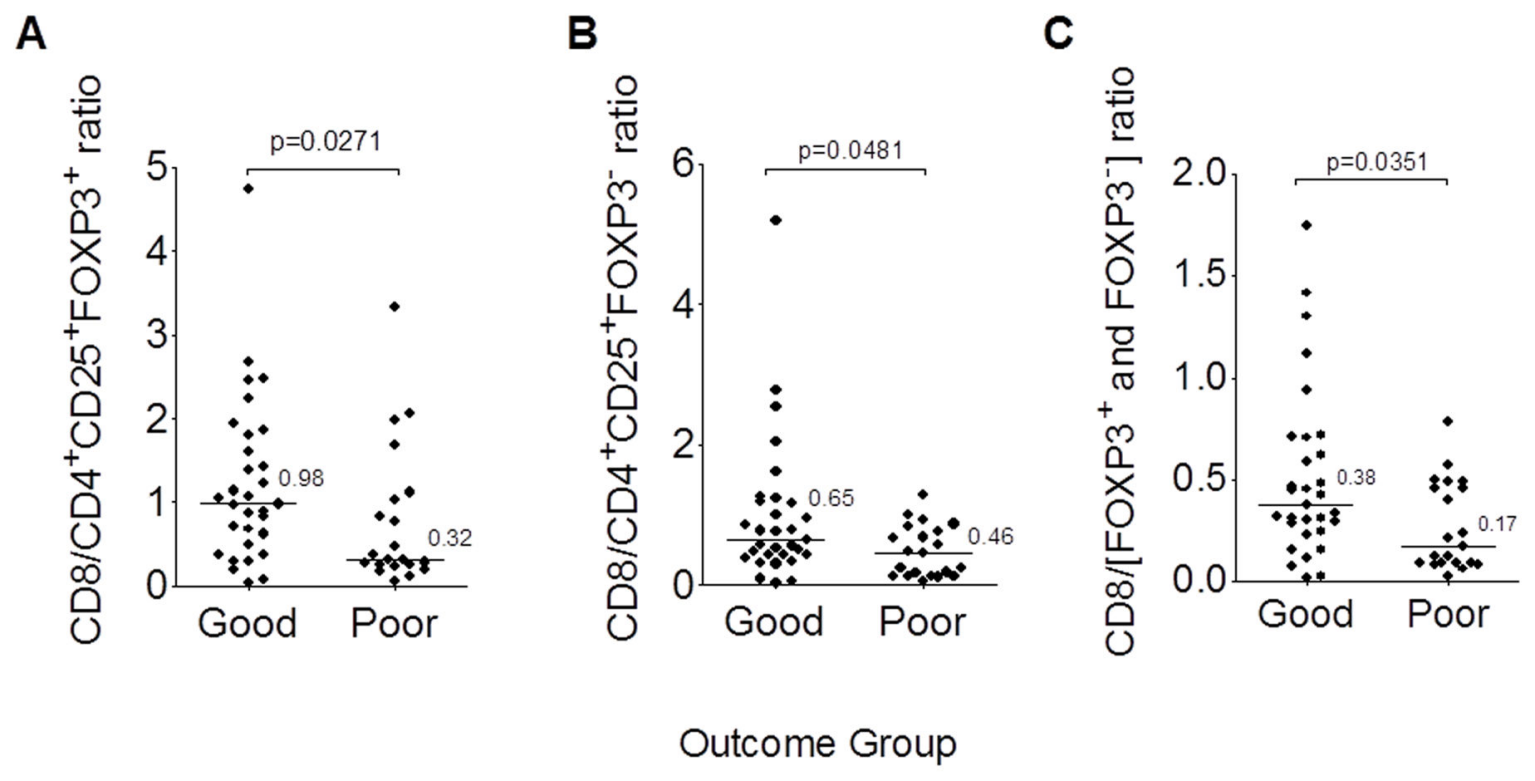
The CD8^+^/Treg ratio correlates with clinical outcome in serous ovarian cancer. (A) Pie chart showing the distribution of CD4^+^ T cells with respect to CD25 and FOXP3 staining in all patients. (**B**-**D**) Scatter dot plots of the ratios of CD8^+^ T cell counts to Tregs (CD4^+^CD25^+^FOXP3^+^) (**B**), CD4^+^CD25^+^FOXP3^-^ T cells (**C**), or combined CD4^+^CD25^+^FOXP3^+^ and CD4^+^CD25^+^FOXP3^-^ T cells (**D**) for both the good outcome and poor outcome groups.

Seeing no associations between the absolute counts of CD4^+^CD25^+^FOXP3^+^ Tregs and clinical outcome, we focused on ratios as had been previously described [8,28]. The median CD8^+^/CD4^+^CD25^+^FOXP3^+^ Treg ratio was found significantly higher in patients with good outcome compared with the patients with poor outcome (0.98 vs. 0.32, p=0.027, Table 2, Figure 3A). In contrast, the median CD3^+^/CD4^+^CD25^+^FOXP3^+^ Treg and CD4^+^/CD4^+^CD25^+^FOXP3^+^ Treg ratios were not significantly different among the groups. Upon further examination, unexpectedly we also detected that the good outcome patients had a median CD8^+^/CD4^+^CD25^+^FOXP3^-^ ratio that was significantly higher in the good group (0.65) as compared to the poor group (0.46, p=0.048) (Table 2, Figure 3B). When the CD8^+^/CD4^+^CD25^+^FOXP3^+^ Treg and CD4^+^CD25^+^FOXP3^-^ ratios were considered together (i.e. all CD4^+^CD25^+^) and as shown in Figure 3C, the ratios between the two groups remained significantly different. As age was found to be slightly different between the two groups, we verified that the association between the cell counts and group status remained consistent after adjusting for age in a logistic regression model (data not shown). 

When comparing the ratios calculated from the three major stains (CD3, CD4, and CD8), only the median CD8^+^/CD4^+^ T cell ratios, which were 0.27 and 0.13 respectively for the good and poor outcome groups, were statistical significantly different (p=0.050), which is consistent with the significant ratios of the CD8^+^ T cells to either the CD4^+^CD25^+^FOXP3^+^ Tregs or the CD4^+^CD25^+^FOXP3^-^ described above. No other ratios (e.g. CD8^+^/CD3^+^ T cell and CD4^+^/CD3^+^ T cell ratios) significantly differed between the good and poor outcome groups (Table 2). 

**Figure 4:**
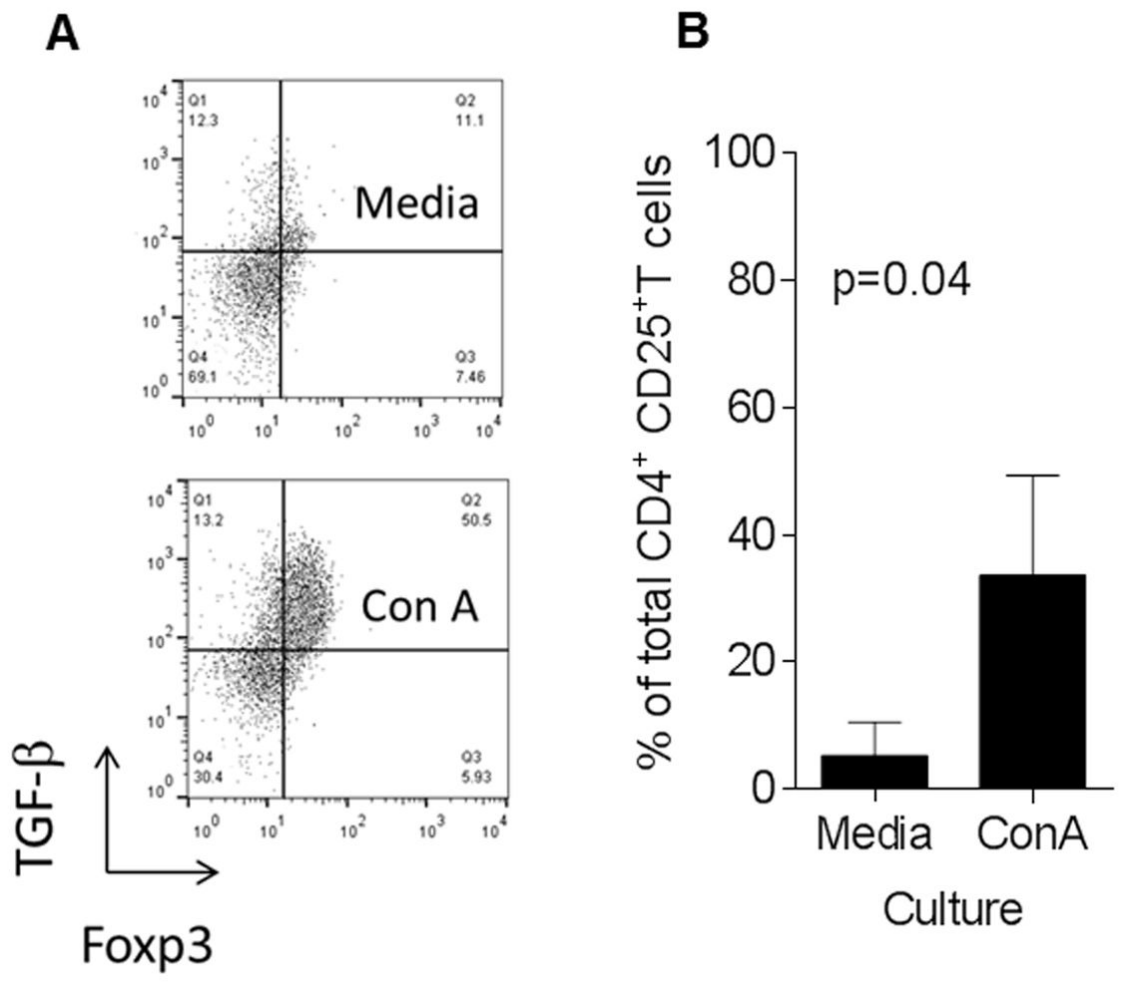
Tumor-infiltrating CD4^+^CD25^+^ cells up-regulate FoxP3 and TGF-β expression upon activation. (**A**) Shown are two dots plots of purified tumor-infiltrating CD4^+^ T cells stimulated with ConA or media alone. Cells are gated on CD4 and CD25. (**B**) Bar charts showing the mean (± SEM, *n*=3 unique patient samples) percentage of FoxP3^+^ and TGF-β T cells among the total CD4^+^CD25^+^ T cells stimulated with either ConA or media alone. P value obtained by a paired Student’s T test.

Lastly, having seen that both FOXP3^+^ and FOXP3^-^ CD4^+^ T cells were associated with outcome when examined as a ratio with CD8^+^ T cells, we speculated that these cells were Tregs capable of producing the immune regulatory cytokine, TGF-β. To test this, we purified tumor-infiltrating leukocytes (TIL) and stimulated then directly *ex vivo* with ConA, followed by intracellular cytokine staining. As shown in Figure 4A, TILs derived CD4^+^CD25^+^ T cells were a mixture of FOXP3^+^ and FOXP3^-^ T cells. In the absence of stimulation, a fraction (12 ± 4%, n=3, ±SEM) of the purified T cells maintained expression of FOXP3. Upon stimulation, the percentage of CD4^+^CD25^+^ T cells expressing FOXP3 increased to 42 ± 9% of the total CD4^+^CD25^+^ T cells. In the absence of stimulation, 5 ± 3% of CD4^+^CD25^+^ T cells produced TGF-β and following stimulation, this increased to 34 ± 9% (p=0.04, Figure 4B). 

## Discussion

Understanding the pathologic role of Tregs that have the ability to suppress activated T cells in the ovarian cancer microenvironment is important for developing new immune-based therapies and determining patient prognosis. In the present study, we found for the first time that CD4^+^CD25^+^FOXP3^+^ Tregs are associated with outcome specifically in serous ovarian cancer but only in the context of CD8^+^ T cells. The fact that the influence of Tregs is only detectable when evaluated as a ratio with cytotoxic CD8^+^ T cells is consistent with the model that potentially destructive tumor-specific immune responses are counterbalanced in the tumor microenvironment by strong immune suppression [29]. This would suggest that strategies aimed at depletion of Tregs and concomitant vaccine stimulation of effector T cells would be effective in eliminating ovarian cancers and improving survival [30]. Several drugs that are known to be useful as Treg-depleting agents such as cyclophosphamide, anti-CD25 antibody, or denileukin diftitox are available for combination therapy with novel vaccine or adoptive T cell therapy approaches [31,32]. Alternatively, Quezada and colleagues show that checkpoint anti-CTLA-4 blockade in combination with vaccine therapy could also be effective at altering the balance without eliminating Tregs, suggesting a potential usage for the recently approved anti-human CTLA-4 antibody in ovarian cancer [33,34].

Our *ex vivo* experiments showed that a low percentage of non-stimulated CD4^+^CD25^+^ T cells maintained FOXP3 expression, which later increased upon activation. This lower frequency of FOXP3^+^ T cells, relative to the immunofluorescence staining results, may reflect down regulation of FOXP3, consistent with observations that human FOXP3 expression is regulated by T cell activation and not developmentally programmed as it is in murine Tregs [35]. Consequently, the expression of FOXP3 in T cells following activation has led to some controversy regarding whether FOXP3 is a Treg-specific marker in humans. Indeed, some studies have shown that FOXP3 expression is elicited in activated T cells with no regulatory activities, which could lead one to speculate that some of the CD4^+^CD25^+^FOXP3^+^ T cells in ovarian cancer may not be regulatory. In contrast, other studies have demonstrated that activated T cells that up-regulate FOXP3 do indeed convey suppressive activity in a cell-contact or cytokine dependent manner (i.e., IL-10 and TGF-β) [36,37]. Although more data are needed, a recent study has provided some clarity showing that FOXP3 localizes primarily in the nucleus of Tregs whereas in activated non-regulatory T cells its localization is cytoplasmic [38]. Thus, with respect to the present study, we focused exclusively on intranuclear FOXP3 suggesting that we evaluated a population enriched in T cells with regulatory activity.

In addition, we observed that CD4^+^CD25^+^FOXP3^-^ T cells are also, albeit more modestly, associated with outcome in an indirect manner that has not previously been reported in the literature. While this phenotype could represent activated Th1 cells, they could also potentially be inducible Tregs in the making, which can acquire regulatory capabilities upon stimulation consistent with our *ex vivo* results showing upregulation of both FOXP3 and TGF-β [39]. As demonstrated in previous studies, the presence of TGF-β during activation is a potent inducer of FOXP3 expression in CD4^+^ non-regulatory T cells, conferring them similar functional characteristics as natural Tregs (i.e., suppressive activity) [40–42]. Siddiqui and colleagues recently reported that, in contrast to expectations, CD4^+^CD25^+^FOXP3^+^ T cells were not significantly associated with renal cell cancer death. Rather, CD4^+^CD25^+^FOXP3^-^ T cells, which produced high levels of IL-10, were significantly associated with outcome [43]. Thus, like renal cell cancer, ovarian cancer may directly induce Tregs in the local microenvironment to prevent the immune system from eradicating the malignancy. 

The lack of direct association of CD4^+^CD25^+^FOXP3^+^ Tregs with outcome is in contrast to the report by Curiel and colleagues whose study showed rather large survival differences associated with infiltration of CD4^+^CD25^+^ T cells which would include both FOXP3^+^ and FOXP3^-^ cells [14]. Specifically, in that study, they found that patients with high levels of tumor associated CD4^+^CD25^+^ T cells had a median survival of 10-20 months whereas those with medium or low levels had 40-50 and 60-70 median survival times, respectively. The major distinctive difference between the present study and Curiel’s, which likely explains the observed discrepancies, is the heterogeneity in the population. In the Curiel study, a mixture of tumor histologies were used in addition to serous including mucinous, endometrioid, clear cell, and undifferentiated, while we used a homogenous sample set consisting only of serous adenocarcinomas. Delineation of the role of Tregs or other lymphocyte populations in ovarian cancer in the future will likely have to account for differences in biology and importantly, survival times among the different histologic subtypes [44]. 

We also showed that CD3^+^ infiltrating T cells alone were not associated with clinical outcome in serous ovarian cancer, which contradicts the Zhang study [7,8]. While we detected CD3^+^ T cell infiltration in all specimens studied, Zhang and colleagues, in contrast, did not detect CD3 staining in 39% of their specimens, which could be due to the sensitivity of the approaches (immunoperoxidase vs. immunofluorescence) used. Some studies have shown that T cell activation triggers rapid CD3/TCR degradation after antigen stimulation and the re-expression of the CD3 molecule is much lower in intraepithelial lymphocytes [26,27]. These might be a few of the reasons why some infiltrating T cells may not be as easily detectable using some techniques. Our analysis showed that the CD3^+^ T cell counts were lower than the CD4^+^ cell count supporting the notion that CD3 staining may not detect all T cells. Similarly, we were unable to replicate the association of intraepithelial CD8^+^ T cells with survival as was seen in the Sato study [8]. The primary difference is the unbiased counting approach we used, in contrast to Sato and colleagues, specifically selecting enriched areas and counting fewer fields (five in their study vs. 20 in ours), which likely led to differences in estimating the numbers of intraepithelial CD8^+^ T cells. 

In conclusion, the general thinking is that serous ovarian cancer evades anti-tumor immunity by investing heavily in an immune suppressive network. One of those mechanisms is thought to be specific recruitment of Tregs. Our study provides new evidence that CD4^+^CD25^+^FOXP3^-^ and CD4^+^CD25^+^FOXP3^+^ T cells constitute part of that network.
